# Identifying new sex-linked genes through BAC sequencing in the dioecious plant *Silene latifolia*

**DOI:** 10.1186/s12864-015-1698-7

**Published:** 2015-07-25

**Authors:** N Blavet, H Blavet, A Muyle, J Käfer, R Cegan, C Deschamps, N Zemp, S Mousset, S Aubourg, R Bergero, D Charlesworth, R Hobza, A Widmer, GAB Marais

**Affiliations:** Institute of Integrative Biology (IBZ), ETH Zurich, Zurich, Switzerland; Institute of Experimental Botany, Centre of the Region Haná for Biotechnological and Agricultural Research, Olomouc - Holice, Czech Republic; Department of Plant Developmental Genetics, Institute of Biophysics of the CAS, v.v.i., Brno, Czech Republic; Laboratoire de Biométrie et Biologie Evolutive (UMR 5558), CNRS/Université Lyon 1, Villeurbanne, France; Pole Rhone-Alpes de Bioinformatique (PRABI), Villeurbanne, France; Unité de Recherche en Génomique Végétale (UMR 1165), INRA/Université d’Evry-Val-d’Essonne – ERL CNRS 8196, Evry, France; Institute of Evolutionary Biology, University of Edinburgh, Edinburgh, UK

**Keywords:** Sex chromosomes, Sex-linked genes, Plant, BAC, RNA-seq, Gene loss, Y degeneration, *Silene latifolia*, *Silene vulgaris*

## Abstract

**Background:**

*Silene latifolia* represents one of the best-studied plant sex chromosome systems. A new approach using RNA-seq data has recently identified hundreds of new sex-linked genes in this species. However, this approach is expected to miss genes that are either not expressed or are expressed at low levels in the tissue(s) used for RNA-seq. Therefore other independent approaches are needed to discover such sex-linked genes.

**Results:**

Here we used 10 well-characterized *S. latifolia* sex-linked genes and their homologs in *Silene vulgaris*, a species without sex chromosomes, to screen BAC libraries of both species. We isolated and sequenced 4 Mb of BAC clones of *S. latifolia* X and Y and *S. vulgaris* genomic regions, which yielded 59 new sex-linked genes (with *S. vulgaris* homologs for some of them). We assembled sequences that we believe represent the tip of the Xq arm. These sequences are clearly not pseudoautosomal, so we infer that the *S. latifolia* X has a single pseudoautosomal region (PAR) on the Xp arm. The estimated mean gene density in X BACs is 2.2 times lower than that in *S. vulgaris* BACs, agreeing with the genome size difference between these species. Gene density was estimated to be extremely low in the Y BAC clones. We compared our BAC-located genes with the sex-linked genes identified in previous RNA-seq studies, and found that about half of them (those with low expression in flower buds) were not identified as sex-linked in previous RNA-seq studies. We compiled a set of ~70 validated X/Y genes and X-hemizygous genes (without Y copies) from the literature, and used these genes to show that X-hemizygous genes have a higher probability of being undetected by the RNA-seq approach, compared with X/Y genes; we used this to estimate that about 30 % of our BAC-located genes must be X-hemizygous. The estimate is similar when we use BAC-located genes that have *S. vulgaris* homologs, which excludes genes that were gained by the X chromosome.

**Conclusions:**

Our BAC sequencing identified 59 new sex-linked genes, and our analysis of these BAC-located genes, in combination with RNA-seq data suggests that gene losses from the *S. latifolia* Y chromosome could be as high as 30 %, higher than previous estimates of 10-20 %.

**Electronic supplementary material:**

The online version of this article (doi:10.1186/s12864-015-1698-7) contains supplementary material, which is available to authorized users.

## Background

Of only a handful of plant sex chromosome systems that have been investigated at the molecular level, the XY chromosome system of *Silene latifolia* is one of the best-studied [1, 2]. However, finding sex-linked genes in this species has been a slow process and is still ongoing. Approaches such as screening cDNA libraries with probes from microdissected *S. latifolia* Y chromosomes identified only a few sex-linked genes (reviewed in [3]). Segregation analysis of intron variants and SNPs within plant families revealed more sex-linked genes (*e.g.* [4, 5]). Altogether, these approaches yielded about 30 validated *S. latifolia* sex-linked genes.

Recently, however, three studies used RNA-seq to identify hundreds of *S. latifolia* sex-linked genes, either using segregation patterns within families [6, 7] or male and female full siblings from an inbred population [8]. Sex-linked genes were identified either by following allele transmission from parents to their progeny (in the two studies using families, [6, 7]), or by searching for SNPs homozygous in females and heterozygous in males, indicating Y-linkage [8]. As no *S. latifolia* reference genome is available, these searches started with either a *de novo* assembled reference transcriptome using the *S. latifoli*a RNA-seq data [7, 8] or using 454 EST data from *S. vulgaris*, a close relative without sex chromosomes [6, 9], to map the *S. latifolia* reads and perform SNP-calling. Both approaches are subject to errors, especially when sex-linkage of a contig is inferred from the segregation pattern of only a single SNP, so the inferences were assessed by checking for complete sex-linkage of some of the inferred sex-linked genes, using PCR on sets of unrelated males and females [6, 7]. Further tests were done to check whether “tester sets” of well-validated sex-linked and autosomal genes (see above) were correctly assigned [6–8]. The results were encouraging, with most genes tested being correctly assigned. However, only a few newly inferred genes (~10 in each study) were checked experimentally, and the tester sets included only 10–20 sex-linked and 0-10 autosomal genes. Moreover, the RNA-seq studies focused on RNA from only one tissue (flower buds) and any sex-linked genes not expressed in flower buds, or expressed at low levels, must be missed [6–8].

The number of sex-linked genes in *S. latifolia* is therefore not yet accurately known. An alternative approach to discovering new sex-linked genes is to sequence BAC clones from the sex chromosomes. A handful of BACs from the *S. latifolia* X and Y chromosomes have already been sequenced (*e.g.* [10, 11]), and they yielded few new sex-linked genes. To improve the yield, we screened a BAC library with probes from validated X-linked or Y-linked genes of *S. latifolia*, which establishes sex-linkage of all genes found in the BAC sequences. Identifying both X-linked and Y-linked genes is important for estimating the proportion of X-linked genes that have lost their Y counterparts, indicating Y genetic degeneration of this plant sex chromosome system. Sequencing BACs should help identify genes with low expression levels, some of which were probably missed by previous studies, because most sex-linked genes identified so far in *S. latifolia* come from cDNA, ESTs or RNA-seq data, which will be enriched for highly expressed genes. Sequencing the complete *S. latifolia* sex chromosomes using BACs would be extremely costly as the X is 400 Mb, and the Y 550 Mb. However, BAC sequencing to obtain sequences of portions of the sex chromosomes is very useful. In particular, it can provide larger tester set to compare with results from RNA-seq studies (see above), as well as for analyses (explained below) for estimating changes in gene densities during the evolution of the X and Y chromosomes, and gene losses from the Y chromosome.

We obtained ~4 Mb of BAC sequences from the *S. latifolia* sex chromosomes and from *Silene vulgaris,* a closely related non-dioecious plant without sex chromosomes, in order to identify both new sex-linked genes and their *S. vulgaris* homologs, which can serve as outgroup sequences for comparing the evolution of *S. latifolia* X-linked and Y-linked genes. A BAC library from a *S. latifolia* male was screened using probes specific for X-linked and Y-linked alleles of 10 previously validated X/Y gene pairs (see Methods and Additional file 2: Table S1). Orthologs of all 10 genes have been identified in *S. vulgaris*, all mapping to a single linkage group [5, 12], indicating that they were all on the ancestral proto-sex chromosomes, and not gained during the evolution of the *S. latifolia* sex chromosomes. Their map locations in *S. latifolia* indicate that they represent all evolutionary strata (chromosomal regions with different levels of X-Y divergence) previously described for this species [5, 13] (see also Additional file 1: Figure S1A). Annotation of the BAC sequences yielded 49 new X-linked genes and 10 new Y-linked genes. We analysed the gene densities of the X-linked, Y-linked and *S. vulgaris* BACs. We also searched by Blast the previously published RNA-seq data with the sequences of the new sex-linked genes in the BACs, and used the results to develop a new, combined approach to estimate Y gene loss. The results of our re-evaluation suggests that gene loss may have been underestimated based on RNA-seq alone, although more work is still needed to get a precise estimate of Y gene loss in *S. latifolia*.

## Results and discussion

### Obtaining *S. latifolia* X and Y genomic sequences and identifying genes

A total of 25 positive BAC clones were selected and sequenced (see Methods, Additional files 2 and 3: Tables S1 and S2). After further validation (see Methods), 24 clones were retained for analysis. These included 6 triplets of X/Y/*vulgaris* sequences, one X/*vulgaris* pair, one Y/*vulgaris* pair, and two single X BAC clones without Y chromosome or *S. vulgaris* homologs (Additional file 2: Table S1). The 16 sex-linked chromosomal fragments sequenced total ~2.5 Mb, the largest set of *S. latifolia* sex-linked genomic sequences so far obtained. These BAC sequences were assembled and annotated (see Methods, Additional files 2 and 3: Tables S1 and S2), revealing a total of 153 genes, 78 of which are from *S. vulgaris*. Including the probe genes, the *S. latifolia* genes total 58 X-linked and 17 Y-linked genes (Table 1 and Additional files 2 and 3: Tables S1 and S2). 59 of them are newly identified in *S. latifolia*, tripling the number of *S. latifolia* fully sex-linked genes with complete genomic sequences; 49 of these 59 new sex-linked genes are X-linked, and 10 are Y-linked.

**Table 1:** Gene number and density in *S. latifolia* X and Y and in *S. vulgaris* BAC clones

	*S. latifolia* X	*S. latifolia* Y	*S. vulgaris*
Total of all genes, including genes used as “probes”	58	17	78
Total number of new genes	49	10	70
Total number of *S. latifolia* / *S. vulgaris* homologous gene pairs	13	2	-
Total physical size (Mb)	1.7	1.09	1.05
Gene density per Mb	34	16	74

Gene density was computed using all available BAC data. When only triplets are used, the results are similar

An all-against-all Blast search among the BAC-located genes revealed conserved blocks of several tens of kb around each probe gene in the *S. latifolia* X and *S. vulgaris* BAC sequences (Additional file 1: Figure S2). These blocks include 13 new X-*vulgaris* homologous gene pairs (Table 1 and Additional file 3: Table S2). When aligning X-linked and *S. vulgaris* sequences using MAUVE (Methods), we found conserved gene orders in the blocks around the probe genes, and sequence similarities in the intergenic regions. In contrast, Blast searching found only two new Y-*vulgaris* gene pairs (Table 1 and Additional file 3: Table S2), and MAUVE alignments found similarity between Y and *S. vulgaris* sequences mostly restricted to the probe gene itself (Additional file 1: Figure S2). This suggests the occurrence of insertions, deletions and other chromosomal rearrangements of the *S. latifolia* Y chromosome at a small (within BAC) scale, in addition to the large-scale rearrangements previously found [13–21].

To directly evaluate the extent of gene losses from the *S. latifolia* Y chromosome, we first searched for X/Y gene pairs (often called “gametologous pairs”, in which X and Y genes are alleles that diverged since X-Y recombination became suppressed), where one is clearly recognizable as a pseudogene. We found no such pairs. All pseudogenes found in the BAC sequences were duplicates of other genes in the same BAC clone. The only X/Y gene pairs in our BAC sequences are the “probe” genes, which were already known (Additional file 3: Table S2); none of the new X-linked genes have gametologs in the corresponding Y chromosome BAC sequence (Additional file 3: Table S2).

### Assembling BACs from the X4, X7 and X6a regions and implications for the number of pseudoautosomal regions in *S. latifolia* sex chromosomes

We found overlaps between the X BAC sequences from three probes, genes X4, X7 and X6a. These BAC sequences were therefore assembled into a scaffold (Additional file 1: Figure S1B). The end of this scaffold (BAC clone BAC65P13) consists of X43.1 repeats typical of *Silene* telomeres [22]. These X43.1 repeats probably represent the X telomere, based on the following reasoning. BAC assembly and sequencing statistics indicate that 7 % of reads in BAC65P13 are from X43.1, yielding an estimate that the X43.1 repeat forms a ~6 kb region of this BAC. No interstitial X.43.1 signal was detected on the X chromosome in previous work using FISH [18], but a 6 kb sequence composed of units arranged in tandem should yield a clear fluorescent signal with the X43.1 probe. A non-telomeric location is therefore unlikely. Our results therefore suggest that we have reached the end of the Xq arm in *S. latifolia.* In turn, this implies that only the Xp end is pseudoautosomal. Our results are therefore consistent with the *S. latifolia* sex chromosomes having only a single pseudoautosomal region, and not two as AFLP mapping suggested [23]; a single pseudoautosomal region (PAR) is consistent with the latest genetic mapping [5] (although our work and [5] do not completely agree on the gene content of the Xq end).

### Gene densities in *S. latifolia* X, Y and *S. vulgaris* BAC clones

We found an average of 34 genes/Mb in the *S. latifolia* X BAC sequences and 74 genes/Mb in those from *S. vulgaris* (Table 1). The gene densities we observed in both species’ BAC sequences are quite high, which suggests that we have sequenced gene-dense regions. The 2.2-fold lower gene density in the *S. latifolia* X is, however, consistent with the expectation based purely on the genome sizes of the two species (2.7 Gb for *S. latifolia* and the 1 Gb for *S. vulgaris*; see the Plant DNA C-value Database, http://data.kew.org/cvalues/). Assuming the same total number of genes in both species (which is likely as they are closely related species with an identical chromosome number of 2n = 24), and neglecting possible inter-chromosomal translocations in *S. latifolia* or *S. vulgaris* [5], the relative total genome sizes predict a 2.7-fold lower gene density in *S. latifolia*.

In contrast, the *S. latifolia* Y BACs have an estimated average gene density of only 16 genes/Mb (Table 1), 2.1 times lower than the X. The *S. latifolia* Y chromosome is 550 Mb, considerably larger than the X (400 Mb; see [24]). If the number of genes were the same on both sex chromosomes (that is, if their size difference is due solely to the accumulation on the Y of sequences not present on the X, including transposable elements, NUMTs and NUPTs [14, 16, 18, 19, 21], and ignoring the possibility that the PAR may represent physically large regions [5]), the ratio of gene densities for Y versus X should be the same as the ratio of Y/X chromosome sizes, 550/400, predicting a mean Y density 1.4 times lower than that of the X. The observed value in the *S. latifolia* Y BAC sequences is nevertheless considerably lower than the expectation, and suggests losses of as much as 34 % of genes from the Y.

### Searching for the BAC-located genes in RNA-seq data

We blasted our BAC-located genes to the RNA-seq contigs from previous studies (see Methods), which produced significant matches for 54 out of 63 genes (Table 2 and Additional file 1: Table S3), showing that most of our BAC-located genes (~85 %) are expressed in flower buds. Only half of these genes were identified as sex-linked by any of the previous studies (Table 2). As predicted (see Background) the genes not detected as sex-linked in any of the RNA-seq studies have much lower expression levels (as estimated by [8]) than those where sex-linkage was detected (RPKM values: 3008.3 versus 11251.2, respectively; the difference is significant by a one-tailed Student’s *t* test, *p*-value = 0.004). This suggests that failure to ascertain genes as sex-linked when they have low expression affects inferences using RNA-seq, in addition to absence of expression of some genes in flower buds.

**Table 2:** Comparison of BAC and RNA-seq data

	Total number	BAC-located genes matching RNA-seq contigs	BAC-located genes matching X/Y-inferred RNA-seq contigs	BAC-located genes matching X-hemizygous-inferred RNA-seq contigs	Sources of RNA-seq data
X-linked BAC-located gene	52	44	12	-	M2012
		36	5	2	BC2011
		31	13	5	CF2011
		46	19	5	All 3 combined
Y-linked BAC-located gene	11	7	3	-	M2012
		6	1	1	BC2011
		6	1	3^a^	CF2011
		8	3	1	All 3 combined
All BAC-located genes	63	54	22	6	All 3 combined

All BAC-located genes are included except the six probe genes for which both X and Y copies were already available. M2012: Muyle *et al.* 2012 (ref. [8]), BC2011: Bergero, Charlesworth 2011 (ref. [6]), CF2011: Chibalina, Filatov 2011 (ref. [7])

^a^Among those 3, two genes were found to be X-hemizygous in [7], and XY in [6, 8]. In the combined data (see details in Methods), we considered these genes to be XY

### Re-evaluating Y gene loss using both BAC and RNA-seq data

Two RNA-seq studies have used X-linked genes to estimate Y gene loss in *S. latifolia*. Only 10 to 20 % of X-linked genes were estimated to have no Y transcripts, suggesting that Y degeneration and male hemizygosity may be modest in *S. latifolia* [6, 7]. Correct inference of X-hemizygous genes is critical for reliably estimating Y gene loss. If the Y copy of an X/Y gene pair is not expressed, or is expressed at low levels in the tissue(s) used for RNA-seq analysis, hemizygosity will be incorrectly inferred and gene losses from the Y will be overestimated. We found some examples of this when comparing the BAC and RNA-seq data (using stringent Blast criteria, see Methods). Two BAC-located genes matched contigs inferred as X/Y gene pairs from one study but with contigs inferred as X-hemizygous in others, and one Y-linked gene matched a contig inferred as X-hemizygous (Table 2).

Among our X-linked BAC-located genes, five matched contigs inferred to be X-hemizygous (Table 2). Using our BAC-located genes that match RNA-seq contigs detected as sex-linked, this yields an estimate of 20 % of Y gene loss, the same as in the published RNA-seq studies [6, 7]. However, if coverage is low due to a low expression level, SNPs may not be identified; individuals cannot then be genotyped and no inferences about sex-linkage are possible. Recent data from animals suggests that average expression levels are lower for X-hemizygous genes than for X/Y gene pairs [25, 26], and therefore the RNA-seq approach may fail to detect X-hemizygous genes more often than X/Y gene pairs, resulting in an underestimation of gene losses from the Y. If this bias occurs, the BAC-located genes not matching contigs inferred as sex-linked should include more X-hemizygous genes than the ~20 % estimate above.

To evaluate this possibility, it would be helpful to have an estimate of the proportion of X-hemizygous genes that were undetected by the RNA-seq studies. When these studies were done, very few validated X-hemizygous genes were available in *S. latifolia*. Only two fully degenerated Y-linked genes in *S. latifolia* have so far been documented [27, 28]. Two recent studies used segregation analysis in large families and inferred further X-hemizygous genes, one being a segregation analysis using RadSeq data [5, 29]; however  comparing these genes with the sex-linked contigs from RNA-seq studies reveals that ~57 % might be X/Y gene pairs, so we cannot use them as well-validated X-hemizygous genes (see the list of genes with X-hemizygous segregation patterns in Additional file 4: Table S4). 

We therefore used an indirect approach. Many well-validated X/Y gene pairs are now available, and can be used to estimate the probability that the combined RNA-seq studies fail to detect such a gene pair. Given this estimate, one can infer how many of the BAC-located genes that do not match sex-linked RNA-seq contigs could represent such missed X/Y gene pairs, and thus how many are probably truly X-hemizygous genes (schematized in Additional file 1: Figure S3). For the required estimate, we used all published well-validated X/Y gene pairs: the 17 experimentally validated ones (see references in Additional file 4: Table S4), 20 sex-linked contigs from RNA-seq studies that were validated by PCR [6], and 12 more from a recent segregation analysis [5]. All these are probably highly expressed genes. We added 21 more X/Y gene pairs from the RadSeq study [29], which uses genomic DNA, and can therefore ascertain genes even if their expression levels are low, for a total of 70 tester genes that were previously inferred as sex-linked. 78 % of these genes had significant matches with contigs from at least one of the three RNA-seq studies, implying that they are expressed in flower buds. Genes matching contigs not assigned as sex-linked in one study often matched sex-linked ones in another, so that only around 25 % of true X/Y gene pairs remained undetected in the three RNA-seq studies combined (Additional file 4: Table S4).

This estimated proportion suggests that, out of our total number of 43 new X-linked BAC-located genes expressed in flower buds, 0.25*43 = 10.75 are probably X/Y gene pairs undetected in the combined RNA-seq data. Thus, 10.75 of the 22 BAC-located genes not matching sex-linked RNA-seq contigs (category (iii) in Table 3) are accounted for. This leaves 22 – 10.75 = 11.25 genes that are probably X-hemizygous, but failed to be detected by the RNA-seq studies. Only X-linked genes newly ascertained by our BAC sequencing are “ancestral” genes relevant for estimating gene losses (the probe genes were ascertained through detecting Y-linked variants, and were therefore previously known to have Y copies); there were probably 50 “ancestral” genes in our BAC sequences, 43 X BAC-located genes that lack copies in our Y BACs but have RNA-seq matches, plus the 7 Y-only BAC-located genes with RNA-seq matches (the total is 60 including the probe genes). The estimated number of Y gene losses is then as follows: 5 genes detected as X-hemizygous (category (ii) in Table 3) + 11.25 X-hemizygous genes that failed to be detected by the RNA-seq studies (see above). Dividing by 50 ancestral genes yields 33 % (or 27 % including the probe genes, Table 3). Using a similar approach to estimate gene losses from the X chromosome gives a considerably lower fraction, 5 % (or 4 % including the probe genes), significantly different from the estimate for the Y (Table 3, Fisher’s exact test p-values < 10^−3^ in either case). Estimates of ancestral gene numbers are particularly reliable when an outgroup is used to exclude genes that were gained after the sex chromosomes originated, by duplication and/or relocation onto the X. We therefore repeated this analysis, restricting it to genes with homologs on the *S. vulgaris* BAC sequences (which must have been present on the ancestral proto-sex chromosomes). The results are similar; excluding the “probe” genes, we estimate 34 % gene loss from the Y, and none from the X (Fisher’s exact test p-value = 0.003; see Additional file 1: Table S5, or, including the “probe” genes, 23 % and 0 % Y and X gene loss, respectively; Fisher’s exact test *p*-value < 0.05).

**Table 3:** Analysis of gene loss in X and Y chromosomes combining BAC and RNA-seq data

Categories of genes	X-linked genes	Y-linked genes
All new genes in BAC sequences	49	10
No match to RNA-seq contigs	6	3
Genes retained for analysis	43	7
Category (i): X/Y gene pair results in RNA-seq analysis	16	2
Category (ii): X-hemizygous results in RNA-seq analysis	5	1
Category (iii): Not ascertained as sex-linked by RNA-seq analysis	22	4
Estimated X/Y false negative rate for gene pairs for RNA-seq analysis^a^	25 %	25 %
Expected number of XY pairs undetected in RNA-seq analysis	10.75	1.75
Potential number of X-hemizygous (X0) or Y0 genes undetected in RNA-seq analysis	11.25	2.25
Potential total number of X-hemizygous (X0) or Y0 genes (sum of detected + undetected in RNA-seq analysis numbers above)	16.25	2.25
Potential proportion of X-hemizygous (X0) or Y0 genes^b^	27-33 %	4-5 %

^a^Based on 39 genes previously known to have X-linked and Y-linked copies, see Additional file 4: Table S4

^b^Based on total numbers of potential ancestral genes, either including the probe genes, or excluding them, respectively (see text for details).

Correct estimation of the proportion of X-hemizygous genes among the BAC-located genes depends on the representativeness of the X/Y gene pairs used as tester set. To check further our set of inferred X-hemizygous genes, we searched for genes that were wrongly classified as X-hemizygous, but which were actually X/Y gene pairs whose sequences are so diverged that they assembled into different contigs, one of which (the Y contig) was not detected. RNA-seq contigs representing the Y copies of these X-hemizygous genes should be found only in males. To test for such sequences among the RNA-seq contigs, we examined the BAC-located genes that the published RNA-seq analyses did not ascertain as sex-linked by blasting them against a set of RNA-seq contigs that were found only in males (from [8]). This yielded only between 3 and 5 significant matches (depending on the filtering of the RNA-seq data, see Methods). Thus, very few potentially highly diverged Y copies are present among the RNA-seq contigs; moreover, some of the male-specific contigs may not represent divergent Y copies but may simply be autosomal paralogs specifically expressed in males. The lack of evidence for the existence of many undetected X/Y gene pairs with diverged Y-linked copies agrees with our estimate that no more than 10 of the genes not ascertained as sex-linked by RNA-seq analysis are actually X/Y gene pairs (Table 3).

## Conclusions

Our BAC sequencing effort resulted in 59 new validated sex-linked genes in *S. latifolia*, adding to the 43 already published ones available (listed in Additional file 4: Table S4). Comparing our new genes to sex-linked genes identified by RNA-seq studies shows that failure to ascertain genes as sex-linked when they have low expression is an important limitation of RNA-seq, in addition to non-expression in the flower bud tissues that have been used, illustrating the difficulty of reliably inferring sex-linkage, X-hemizygosity and gene loss from the Y chromosome without a reference genome. Analyses to take this ascertainment bias into account suggest that gene losses from the *S. latifolia* Y could be higher than previously thought, perhaps around 30 %, consistent with the gene densities in X/Y and *S. vulgaris* BACs. However, further work is needed to estimate Y gene loss in this species more precisely.

## Methods

### Isolation and sequencing of BAC clones

The BAC library was screened following [30]. Clones were gridded on nylon membrane filters and hybridized. The *S. latifolia* BAC library includes a total of 119,808 clones, with an average insert-size of 128 kb, which equates to 5.3 times the male haploid genome. The *S. vulgaris* BAC library (total of 55,296 clones), with an average insert-size of 110 kb, represents 6.8 haploid genomes of this species. We screened these libraries using probes designed from 10 published sex-linked genes and their homologs in *S. vulgaris* (shown in Additional file 1: Figure S1A, plus the triplet SlAP3X/Y-SvAP3). For each “probe” gene, the X-linked copy was used to screen the *S. latifolia* BAC library, and the Y copy to identify Y-linked BAC clones in the *S. latifolia* BAC library, while the *S. vulgaris* homolog was used to identify *S. vulgaris* BAC clones. For each probe, we found 1 to >100 positive clones. We selected clones showing strong hybridization with the probe, and only those that were confirmed by PCR with probe-derived primers were used in further analyses. Whenever possible, we sequenced one BAC clone for each probe gene. These clones were sequenced with coverage varying from 5–6 to 8–600 X for Sanger and 454, respectively (some clones with mate-pairs, and some without). The BAC sequences were validated by comparing the sequence of the “probe” gene from the BAC to the published sequence of the “probe” gene; this excluded only one BAC clone. This yielded complete triplets of X, Y and *S. vulgaris* BACs for some probe genes, but not all (Additional file 2: Table S1). All the “probe” genes except *SlAP3* have already been mapped on the *S. latifolia* X chromosomes [4, 5], and their Y copies have been mapped on Y chromosome physical maps, see [13]. All the BAC contigs are available in Genbank (Accession numbers KC978922-KC977838). Additional file 2: Table S1 provides more details.

### Assembly and annotation of BAC sequences

For each BAC clone, the reads were assembled *de novo* using Newbler v.2.5.3 (2010), except for three BAC clones sequenced using Sanger sequencing (19P24, 93 L17 and 78D08), which were assembled with phrap v.16 (2007). The assembly statistics in Additional file 2: Table S1 were obtained using QUAST [31]. Annotation (see Additional file 3: Table S2) was done using both homology-based and expression-data-based strategies using Uniprot and *S. latifolia* RNA-seq data from [8]. Truncated genes and genes with premature stop codons and/or frameshifts were annotated as pseudogenes. DNA repeats (including transposable elements) were annotated using the latest update of the database of DNA repeats in *S. latifolia*, based on an extensive search using genomic library screening and low coverage sequencing of the *S. latifolia* data [18, 20].

### Sequence analysis

Homology among BAC clones from the same X/Y probe gene pair was assessed by aligning the BAC sequences with MAUVE 2.3.1 [32] after masking the repeats using RepeatMasker v3.3.0 (http://www.repeatmasker.org/) with the *Silene* DNA repeat database mentioned above. Homology between X/Y BAC pairs was also assessed by performing an all-against-all Blast search (with the default parameters) among the genes found in the X/Y BAC pair. The results are shown in Additional file 1: Figure S2, and the X-vulgaris and Y-vulgaris pairs that we found are listed in Additional file 1: Table S6.

To obtain the results shown in Table 2, we performed a Blast search of all coding sequences (CDS, obtained by annotating the BAC sequences, see previous section) against the RNA-seq data from the three previous studies [6–8] using data available in Genbank [7] and our own data [6, 8]. We retained only manually checked Blast hits with e-values < 10^−5^, % identities > 90 %, and alignment lengths > 50 bp. Multiple corresponding RNA-seq contigs were allowed for a single BAC CDS to account for assembly problems in the RNA-seq data. The three RNA-seq studies were then combined to infer each CDS gene as being X/Y, X-hemizygous, or not detected as sex-linked in the RNA-seq data (Additional file 3: Table S2). A gene was classified as X/Y in RNA-seq data if any one of the matching RNA-seq contigs was classified as X/Y, and as X-hemizygous if it satisfied two criteria: (i) at least one matching RNA-seq contig was classified as X-hemizygous, and (ii) all other matching RNA-seq contigs were not classified as X/Y gene pairs. Finally, the gene was classified as not having been detected as sex-linked in RNA-seq data whenever all matching RNA-seq contigs failed to be detected as sex-linked. Expression level estimates were obtained from [8].

To check our X-hemizygous genes, we blasted them all (including those detected as X-hemizygous in the RNA-seq studies) against a set of RNA-seq contigs expressed only in males (using data from [8]). Some of these genes might correspond to sex-linked genes with highly diverged X and Y copies that assembled in separate RNA-seq contigs and might therefore be wrongly classified as X-hemizygous, or not be detected as sex-linked at all. To test for potentially Y-linked sequences, we used a set of male-specific contigs from the RNA-seq results. We required these contigs to be expressed in all males and none of the females, using (i) all male-specific contigs, N = 5,504 (ii) male-specific contigs without matches to any transposable element sequence (using the *S. latifolia* TE database mentioned above) and with more than 10 mapped reads in one of the libraries (to remove noisy expression), N = 3,400. Only sequences with Blast hits of > 100 bp, e-values < 10^−4^, scores > 80 and identities > 80 % were retained.

Fisher’s exact tests and Student’s *t* tests were done using the relevant statistical functions in R (http://www.r-project.org/).

### Availability of supporting data

The BAC contigs are available in Genbank (Accession numbers KC978922-KC977838).

## Additional files

Additional file 1: Figures S1. A) Localization of the BAC clones on the *S. latifolia* X and Y chromosomes. **Figure S2.** Annotation of all BAC clones. Blue bars = “probe” genes, black bars = new genes, red triangles = transposable elements. **Figure S3.** Pipeline for inferring Y gene loss. X-linked BAC-located genes are blasted against the RNAseq contigs. **Table S3.** Comparison of BAC and RNAseq data (detailed table). **Table S5.** Analysis of gene loss in X and Y chromosomes using combined BAC and RNAseq data (for X-vulgaris and Y-vulgaris pairs only). **Table S6.** List of new X*-vulgaris* and Y*-vulgaris* pairs. Additional file 2: Table S1. List of BAC clones. Additional file 3: Table S2. List of BAC-located CDS. Additional file 4: Table S4. List of genes in the tester set.

## Abbreviations

BAC: Bacterial Artificial Chromosome
EST: Expressed Sequence Tag
NUMT: Nuclear Mitochondrial DNA
NUPT: Nuclear plastid DNA
PAR: Pseudoautosomal region
RPKM: Reads per Kilobases per Million
SNP: Single Nucleotide Polymorphism

## References

[1] Bernasconi G, Antonovics J, Biere A, Charlesworth D, Delph LF, Filatov D (2009). *Silene* as a model system in ecology and evolution. Heredity.

[2] Ming R, Bendahmane A, Renner SS (2011). Sex chromosomes in land plants. Annu Rev Plant Biol.

[3] Filatov DA (2005). Isolation of genes from plant Y chromosomes. Methods Enzymol.

[4] Bergero R, Forrest A, Kamau E, Charlesworth D (2007). Evolutionary strata on the X chromosomes of the dioecious plant *Silene latifolia*: evidence from new sex-linked genes. Genetics.

[5] Bergero R, Qiu S, Forrest A, Borthwick H, Charlesworth D (2013). Expansion of the pseudo-autosomal region and ongoing recombination suppression in the *Silene latifolia* sex chromosomes. Genetics.

[6] Bergero R, Charlesworth D (2011). Preservation of the Y transcriptome in a 10-million-year-old plant sex chromosome system. Curr Biol.

[7] Chibalina MV, Filatov DA (2011). Plant Y chromosome degeneration is retarded by haploid purifying selection. Curr Biol.

[8] Muyle A, Zemp N, Deschamps C, Mousset S, Widmer A, Marais G. Rapid de novo evolution of X chromosome dosage compensation in *Silene latifolia*, a plant with young sex chromosomes. PLoS Biol. 2012;10(4), e1001308. 10.1371/journal.pbio.1001308 PMC3328428 22529744

[9] Sloan DB, Keller SR, Berardi AE, Sanderson BJ, Karpovich JF, Taylor DR (2012). De novo transcriptome assembly and polymorphism detection in the flowering plant *Silene vulgaris* (Caryophyllaceae). Mol Ecol Resour.

[10] Ishii K, Amanai Y, Kazama Y, Ikeda M, Kamada H, Kawano S (2010). Analysis of BAC clones containing homologous sequences on the end of the Xq arm and on chromosome 7 in the dioecious plant *Silene latifolia*. Genome.

[11] Blavet N, Blavet H, Cegan R, Zemp N, Zdanska J, Janousek B (2012). Comparative analysis of a plant pseudoautosomal region (PAR) in *Silene latifolia* with the corresponding *S. vulgaris* autosome. BMC Genomics.

[12] Filatov DA (2005). Evolutionary history of *Silene latifolia* sex chromosomes revealed by genetic mapping of four genes. Genetics.

[13] Bergero R, Charlesworth D, Filatov DA, Moore RC (2008). Defining regions and rearrangements of the *Silene latifolia* Y chromosome. Genetics.

[14] Pritham EJ, Zhang YH, Feschotte C, Kesseli RV. An Ac-like transposable element family with transcriptionally active Y-linked copies in the white campion *Silene latifolia*. Genetics. 2003;165(2):799–807. 10.1093/genetics/165.2.799 PMC1462803 14573489

[15] Hobza R, Lengerova M, Svoboda J, Kubekova H, Kejnovsky E, Vyskot B (2006). An accumulation of tandem DNA repeats on the Y chromosome in *Silene latifolia* during early stages of sex chromosome evolution. Chromosoma.

[16] Kejnovsky E, Kubat Z, Hobza R, Lengerova M, Sato S, Tabata S (2006). Accumulation of chloroplast DNA sequences on the Y chromosome of *Silene latifolia*. Genetica.

[17] Hobza R, Kejnovsky E, Vyskot B, Widmer A (2007). The role of chromosomal rearrangements in the evolution of *Silene latifolia* sex chromosomes. Mol Genet Genomics.

[18] Cermak T, Kubat Z, Hobza R, Koblizkova A, Widmer A, Macas J (2008). Survey of repetitive sequences in *Silene latifolia* with respect to their distribution on sex chromosomes. Chromosome Res.

[19] Bergero R, Forrest A, Charlesworth D. Active miniature transposons from a plant genome and its non-recombining Y chromosome. Genetics. 2008;178(2):1085–92. 10.1534/genetics.107.081745 PMC2248354 18245352

[20] Macas J, Kejnovsky E, Neumann P, Novak P, Koblizkova A, Vyskot B (2011). Next generation sequencing-based analysis of repetitive DNA in the model dioecious plant *Silene latifolia*. PLoS ONE.

[21] Steflova P, Hobza R, Vyskot B, Kejnovsky E. Strong Accumulation of Chloroplast DNA in the Y Chromosomes of Rumex acetosa and Silene latifolia. Cytogenet Genome Res. 2013; 142(1):59-65. 10.1159/000355212 24051898

[22] Sykorova E, Cartagena J, Horakova M, Fukui K, Fajkus J (2003). Characterization of telomere-subtelomere junctions in *Silene latifolia*. Mol Genet Genomics.

[23] Scotti I, Delph LF (2006). Selective trade-offs and sex-chromosome evolution in *Silene latifolia*. Evolution.

[24] Matsunaga S, Hizume M, Kawano S, Kuroiwa T (1994). Cytological analysis in *Melandrium album*: genome size, chromosome size and fluorescence *in situ* hybridization. Cytologia.

[25] Bellott DW, Hughes JF, Skaletsky H, Brown LG, Pyntikova T, Cho TJ (2014). Mammalian Y chromosomes retain widely expressed dosage-sensitive regulators. Nature.

[26] Cortez D, Marin R, Toledo-Flores D, Froidevaux L, Liechti A, Waters PD (2014). Origins and functional evolution of Y chromosomes across mammals. Nature.

[27] Guttman DS, Charlesworth D (1998). An X-linked gene with a degenerate Y-linked homologue in a dioecious plant. Nature.

[28] Kazama Y, Nishihara K, Bergero R, Fujiwara MT, Abe T, Charlesworth D (2012). SlWUS1; an X-linked gene having no homologous Y-linked copy in *Silene latifolia*. G3 (Bethesda).

[29] Qiu S, Bergero R, Guirao-Rico S, Campos JL, Cezard T, Gharbi T, Charlesworth D. RAD-mapping reveals an evolving, polymorphic and fuzzy boundary of a plant pseudoautosomal region. Molecular Ecology. 2015. in press. 10.1111/mec.13297 26139514

[30] Cegan R, Marais GA, Kubekova H, Blavet N, Widmer A, Vyskot B (2010). Structure and evolution of Apetala3, a sex-linked gene in *Silene latifolia*. BMC Plant Biol.

[31] Gurevich A, Saveliev V, Vyahhi N, Tesler G (2013). QUAST: quality assessment tool for genome assemblies. Bioinformatics.

[32] Darling AE, Mau B, Perna NT (2010). progressiveMauve: multiple genome alignment with gene gain, loss and rearrangement. PLoS ONE.

