# Supplementary material for: Identifying new sex-linked genes through BAC sequencing in the dioecious plant Silene latifolia
Source: BMC Genomics. 2015 Jul 25;16(1):546. doi: 10.1186/s12864-015-1698-7 (PMC4520012; doi:10.1186/s12864-015-1698-7)
Supplement: Additional file 1: Figures S1. — A) Localization of the BAC clones on the S. latifolia X and Y chromosomes. Figure S2. Annotation of all BAC clones. Blue bars = “probe” genes, black bars = new genes, red triangles = transposable elements. Figure S3. Pipeline for inferring Y gene loss. X-linked BAC-located genes are blasted against the RNAseq contigs. Table S3. Comparison of BAC and RNAseq data (detailed table). Table S5. Analysis of gene loss in X and Y chromosomes using combined BAC and RNAseq data (for X-vulgaris and Y-vulgaris pairs only). Table S6. List of new X-vulgaris and Y-vulgaris pairs. [file 12864_2015_1698_MOESM1_ESM.docx]

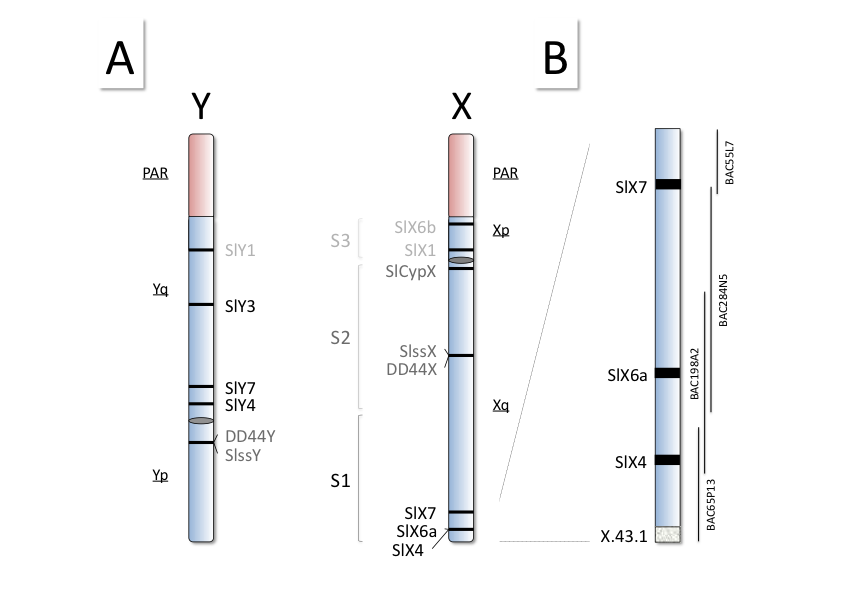


Figure S1: A) Localization of the BAC clones on the *S. latifolia* X and Y chromosomes. Schematic view of the X chromosome genetic map (adapted from (Bergero et al. 2007; Bergero et al. 2013) and of the Y chromosome deletion map (adapted from (Bergero et al. 2008)). The three strata (S1, S2, S3) as defined in (Bergero et al. 2008) are shown on the X chromosome. The positions of the X-linked and Y-linked genes used as probes to screen the *S. latifolia* male BAC library are shown. *SlAP3X* was also used as a probe but mapping data are not available for this gene. Selection and validation of several BAC clones was unsuccessful, which explains why we do not have just BAC triplets (Methods). See Table S1 for a complete list of the sequenced BACs. B) Assembly of the Xq arm. The BACs including *SlX7*, *SlX6a* and *SlX4* are overlapping and we used this to assemble of the end of the Xq arm, where the typical X.43.1 telomeric repeats were found.


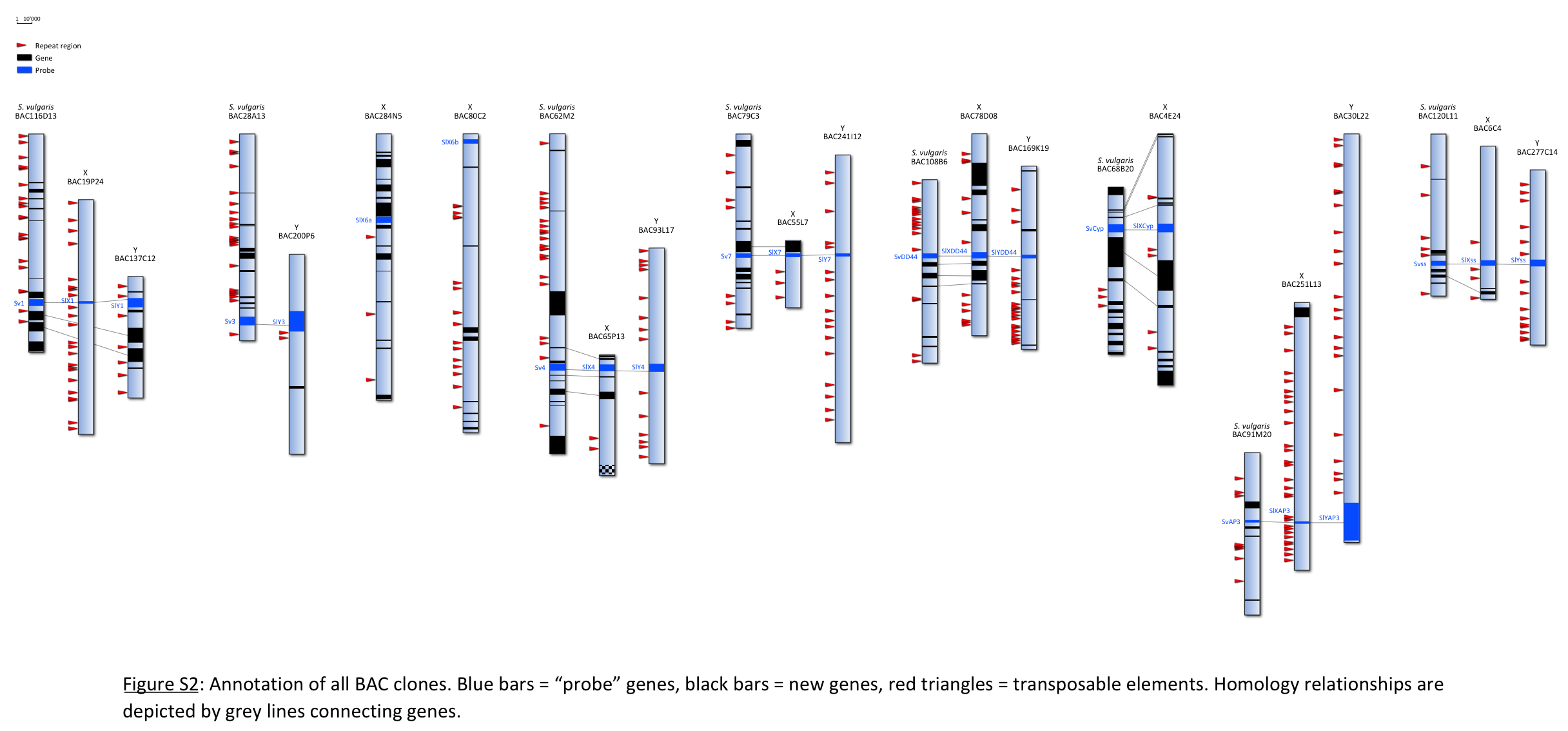


Figure S2: Annotation of all BAC clones. Blue bars = “probe” genes, black bars = new genes, red triangles = transposable elements. Homology relationships are depicted by grey lines connecting genes.


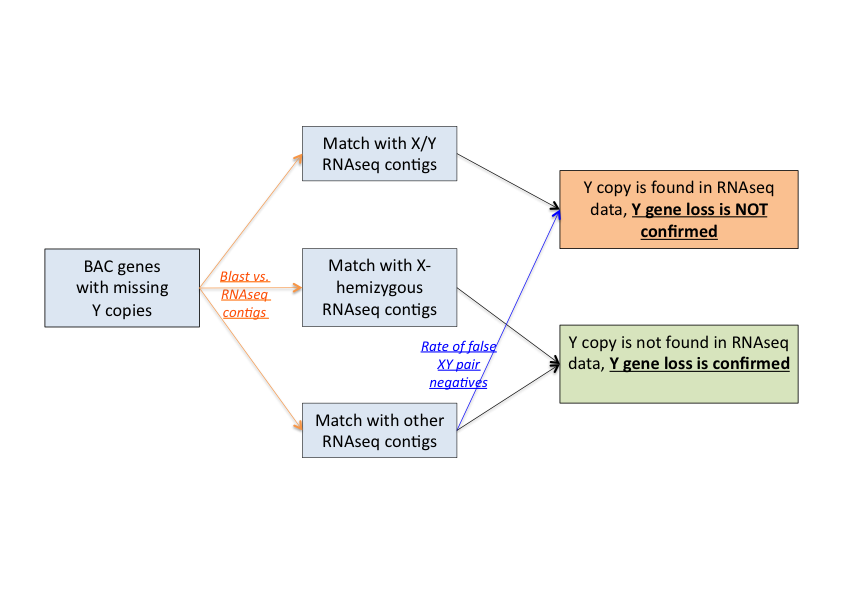


Figure S3: Pipeline for inferring Y gene loss. X-linked BAC-located genes are blasted against the RNAseq contigs. This gives three categories of BAC-located genes: (i) match with a X/Y RNAseq contig, (ii) match with a X-hemizygous contig, (iii) match with an RNAseq contig not detected as sex-linked (see Methods for details about the blast search and how we combined results using different RNAseq datasets). Using the rate of false negatives for X/Y gene pairs, the numbers of undetected X/Y gene pairs and X-hemizygous genes are obtained. This then gives the total number of X-hemizygous genes, which is used to compute the percentage of gene loss on the Y chromosome. Gene loss on the X chromosome is computed similarly using Y-linked BAC-located genes but is less precise, as the Y0 genes are not inferred in RNAseq studies.

Table S3: Comparison of BAC and RNAseq data (detailed table)

|  | BAC-located gene number | BAC-located genes matching RNAseq contigs | BAC-located genes matching X/Y RNAseq contigs | BAC-located genes matching X-hemizygous RNAseq contigs | Sources of RNAseq data |
| --- | --- | --- | --- | --- | --- |
| BAC-located gene with missing Y copy | 52 (15) | 44 (15) | 12 (4) | - | Muyle et al. 2012 |
|  |  | 36 (11) | 5 (4) | 2 (1) | Bergero, Charlesworth 2011 |
|  |  | 31 (8) | 13 (5) | 5 (2) | Chibalina, Filatov 2011 |
|  |  | 46 (15) | 19 (6) | 5 (2) | Combined dataset |
| BAC-located gene with missing X copy | 11 (3) | 7 (3) | 3 (3) | - | Muyle et al. 2012 |
|  |  | 6 (3) | 1 (1) | 1 (0) | Bergero, Charlesworth 2011 |
|  |  | 6 (3) | 1 (1) | 3 (2)^[[1]](#footnote-1)^ | Chibalina, Filatov 2011 |
|  |  | 8 (3) | 3 (3) | 1 (0) | Combined dataset |

Numbers of genes with *S. vulgaris* orthologs are indicated in parentheses

Table S5: Analysis of gene loss in X and Y chromosomes using combined BAC and RNAseq data (for X-vulgaris and Y-vulgaris pairs only)

| Categories of genes | X-linked genes | Y-linked genes |
| --- | --- | --- |
| All new genes in BAC sequences | 15 | 3 |
| No match to RNAseq contigs | 0 | 0 |
| Genes retained for analysis | 15 | 3 |
| Category (i): XY results in RNAseq analysis | 6 | 3 |
| Category (ii): X-hemizygous results in RNAseq analysis | 2 | 0 |
| Category (iii): Not ascertained as sex-linked by RNAseq analysis | 7 | 0 |
| Estimated X/Y false negative rate for gene pairs for RNAseq analysis^[[2]](#footnote-2)^ | 25% | 25% |
| Expected number of XY pairs undetected in RNA-seq analysis | 3.75 | 0.75 |
| Potential number of X-hemizygous (X0) or Y0 genes undetected in RNA-seq analysis | 3.25 | 0 |
| Potential total number of X-hemizygous (X0) or Y0 genes (sum of detected + undetected in RNA-seq analysis numbers above) | 5.25 | 0 |
| Potential proportion number of X-hemizygous (X0) or Y0 genes ^[[3]](#footnote-3)^ | 22-29% | 0% |

**Table S6: List of new X-*vulgaris* and Y-*vulgaris* pairs**

| *S. latifolia* sex-linked BAC-located gene | *S. vulgaris* homolog |
| --- | --- |
| BAC137C12_SlY1_CDS03_AT3G13870.1 | BAC116D13_Sv1_CDS02_AT3G13870.1 |
| BAC137C12_SlY1_CDS05_AT3G13750.1 | BAC116D13_Sv1_CDS07_AT3G13750.1 |
| BAC65P13_SlX4_CDS02_AT2G31410.1 | BAC62M2_Sv4_CDS10_AT2G31410.1 |
| BAC65P13_SlX4_CDS03_AT4G12970.1 | BAC62M2_Sv4_CDS03_AT4G12970.1 |
| BAC65P13_SlX4_CDS04_AT3G26790.1 | BAC62M2_Sv4_CDS01_AT3G26790.1 |
| BAC55L7_SlX7_CDS01_AT3G02050.1 | BAC79C3_Sv7_CDS04_AT3G02050.1 |
| BAC78D08_SlDD44X_CDS07_AT3G63530.1 | BAC108B6_SvDD44_CDS03_AT3G63530.1 |
| BAC78D08_SlDD44X_CDS05_AT3G08505.1 | BAC108B6_SvDD44_CDS05_AT3G08505.1 |
| BAC78D08_SlDD44X_CDS06_AT3G12650.1 | BAC108B6_SvDD44_CDS04_AT3G12650.1 |
| BAC4E24_SlCypX_CDS05_AT3G11590.1 | BAC68B20_SvCyp_CDS09_AT3G11590.1 |
| BAC4E24_SlCypX_CDS06_AT1G08380.1 | BAC68B20_SvCyp_CDS10_AT1G08380.1 |
| BAC4E24_SlCypX_CDS10_AT2G36100.1 | BAC68B20_SvCyp_CDS11_AT2G36100.1 |
| BAC4E24_SlCypX_CDS11_AT1G08260.1 | BAC68B20_SvCyp_CDS01_AT1G08260.1 |
| BAC4E24_SlCypX_CDS07_AT2G27690.1 | BAC68B20_SvCyp_CDS12_AT2G27690.1 |
| BAC6C4_SlssX_CDS01_tr\|A5B1K4\|A5B1K4_VITVI | BAC120L11_Svss_CDS05_tr\|A5B1K4\|A5B1K4_VITVI |

**References**

Bergero R, Charlesworth D, Filatov DA, Moore RC: **Defining regions and rearrangements of the Silene latifolia Y chromosome**. *Genetics* 2008, **178**(4):2045-2053.

Bergero R, Charlesworth D: **Preservation of the Y transcriptome in a 10-million-year-old plant sex chromosome system**. *Curr Biol* 2011, **21**(17):1470-1474.

Bergero R, Forrest A, Kamau E, Charlesworth D: **Evolutionary strata on the X chromosomes of the dioecious plant Silene latifolia: evidence from new sex-linked genes**. *Genetics* 2007, **175**(4):1945-1954.

Bergero R, Qiu S, Forrest A, Borthwick H, Charlesworth D: **Expansion of the pseudo-autosomal region and ongoing recombination suppression in the Silene latifolia sex chromosomes**. *Genetics* 2013, **194**(3):673-686.

Chibalina MV, Filatov DA: **Plant Y chromosome degeneration is retarded by haploid purifying selection**. *Curr Biol* 2011, **21**(17):1475-1479.

Muyle A, Zemp N, Deschamps C, Mousset S, Widmer A, Marais G: **Rapid De Novo Evolution of X Chromosome Dosage Compensation in *Silene latifolia*, a Plant with Young Sex Chromosomes**. *PloS Biol* 2012, **10**(4):e1001308.

1. These two genes were found to be X-hemizygous in (Chibalina, Filatov 2011), but to be XY in (Bergero, Charlesworth 2011; Muyle et al. 2012). In the combined data (see details in Materials and Methods), we considered these genes to be XY [↑](#footnote-ref-1)
2. Based on 39 genes previously known to have X- and Y-linked copies, see Table S4. [↑](#footnote-ref-2)
3. Potential total number of genes absent/number of ancestral genes (including or not the probe genes), see text for details. [↑](#footnote-ref-3)
